# Do movement behaviors identify reproductive habitat sampling for wild turkeys?

**DOI:** 10.1002/ece3.2401

**Published:** 2016-09-14

**Authors:** Mason D. Conley, Nathan A. Yeldell, Michael. J. Chamberlain, Bret A. Collier

**Affiliations:** ^1^ Department of Wildlife and Fisheries Sciences Texas A&M University College Station Texas 77845; ^2^ Warnell School of Forestry and Natural Resources University of Georgia Athens Georgia 30602; ^3^ School of Renewable Natural Resources Louisiana State University Agricultural Center Baton Rouge Louisiana 70803

**Keywords:** Habitat selection, incubation, movements, nest site selection, turkeys

## Abstract

Selection of habitats has regularly been suggested to influence species demography at both local and broad scales. The expectation is that selection behaviors have positive benefits via greater fitness or increased survival. The current paradigm of habitat selection theory suggests a hierarchical process, where an individual first selects where they choose to live (e.g., range) and then searches and selects locations within this range meeting life history needs. Using high‐frequency GPS data collected from reproductively active Rio Grande (*n *=* *21) and Eastern (*n *=* *23) wild turkeys, we evaluated a long‐standing theory for ground‐nesting galliformes, in that movements during the prenesting period are behaviorally focused on sampling available habitats to optimize the selection of nesting sites. Contrary to expectations, we found no evidence that reproductively active females engage in habitat sampling activities. Although most nest sites (>80% for both subspecies) fell within the prenesting range, the average minimum daily distance from nest sites for Rio Grande and Eastern wild turkey females was large [1636.04 m (SE* *=* *1523.96) and 1937.42 m (SE* *=* *1267.84), respectively] whereas the average absolute minimum distance from the nest site for both Rio Grande and Eastern wild turkey females was 166.46 m (SE* *=* *299.34) and 235.01 m (SE* *=* *337.90), respectively, and showed no clear temporal reduction as laying approached. Overall, predicted probability that any female movements before laying were initiated intersected with her nesting range (area used during incubation) was <0.25, indicating little evidence of habitat sampling. Our results suggest that the long‐standing assumption of hierarchical habitat selection by wild turkeys to identify nest sites may be incorrect. As such, habitat selection may not be the proximate driver of nest success and hence population‐level fitness. Rather, based on our results, we suggest that wild turkeys and other ground‐nesting species may be fairly plastic with regard to the selection of reproductive habitats, which is appropriate given the stochasticity of the environments they inhabit.

## Introduction

Patterns of habitat selection have long been suggested to influence species demography at both local and broad scales. In the simplest sense, habitat selection, or the process under which individual behavioral decisions drive use or nonuse of particular habitat types, requires selection to have positive benefits to species demography via greater fitness or increased survival (Jones 2001). Selection is regularly posited as a hierarchical process, where an individual first selects where they choose to live (e.g., range) and then searches and selects locations within this range specific to demographic needs (E. L. Charnov and G. H. Orians, unpubl. data). As such, several authors (Orians and Wittenberger 1991; Badyaev et al. 1996; Jones 2001) have posited that the selection of particular habitat type, specific to life history period, is important to population‐level demography. Adaptive site familiarity provides the foundation that behavioral decisions are driven by familiarity with local conditions, which drives the habitat selection process (Maynard Smith and Parker 1976; Matthiopoulos et al. 2005). Hence, habitat selection theory suggests that decisions on habitat exploration or behavioral searching are primarily made based on features of the landscape, which individuals know or predict will confer positive demographic benefits. The standard expectation is that the longer an individual has to select and evaluate habitat, the more information can be garnered and used to identify optimal locations, such as those used for reproductive activities (Orians and Wittenberger 1991).

A primary driver of avian demography is reproduction, so the selection of nest sites has been of considerable interest (Clark and Shutler 1999; Jones 2001) as the primary limitation to nest success is predation (Sih et al. 1985; Clark and Shutler 1999). Identifying nest sites, to which most individuals will be tied for the duration of the reproductive period, should dominate all other components of habitat selection (Orians and Wittenberger 1991). Habitat sampling, or the process of individuals moving through their range and identifying conditions that should optimize demography, has been suggested to drive unbalanced selection of habitat types (Badyaev et al. 1996). Greater habitat sampling (reflected as area covered prior to nesting) should allow for acquisition and identification of optimal nesting locations. Optimal nesting locations, in turn, should improve safety during incubation under the assumption that habitat effects mediate predation of females or nests (Martin 1993).

Habitat selection by wild turkeys has been well documented (Miller et al. 1999; Thogmartin 1999), and both nest site characteristics (Seiss et al. 1990; Chamberlain and Leopold 1998; Streich et al. 2015) and habitat characteristics within prenesting ranges (Badyaev et al. 1996; Chamberlain and Leopold 2000; Lehman et al. 2008) have been thoroughly described. Most literature suggests that nest site vegetation characteristics provide structural cover that will impede or limit predation (Badyaev 1995; Fuller et al. 2013), although some evidence for reduction in foraging efficiency due to the number of potential locations which could be nest site locations, often denoted as unoccupied prey sites (Charnov 1976, Martin 1993) has been suggested (Chamberlain et al. 2003; Locke et al. 2013; Conley et al. 2015). Additionally, increased movements (e.g., habitat sampling) during the prenesting period has been purported to influence the selection of nest sites (Badyaev et al. 1996; Chamberlain and Leopold 2000; Lehman et al. 2008). For wild turkeys, most of this work has hinged on the process model outlined by Badyaev et al. (1996; also see Badyaev and Faust 1996), wherein wild turkey females select a nest site by narrowing down and selecting habitats using predetermined criteria (e.g., site familiarity). If this assumption holds true, movements would narrow down as females evaluate and identify suitable versus nonsuitable habitat, returning to areas of suitable habitats more frequently until a site is selected and the laying process beings. It is under this assumption that we are focusing our work, using movement behavior as a proxy to site selection as the mechanistic process of selection is unknown.

Habitat sampling, as outlined above, implies that females should select sites in advance of nesting activities and thus should have some ability to accurately predict site suitability in the future (Matthiopoulos et al. 2005). However, landscapes and vegetative conditions occupied by ground‐nesting birds are typically stochastic, regularly impacted by anthropogenic factors such as prescribed fire and environmental factors such as drought/flooding or wildfire (Collier et al. 2009; Oetgen et al. 2015). Therefore, we propose that habitat sampling is unlikely for ground‐nesting birds with a reproductive period that requires between 30 and 40 days to successfully complete. Thus, we evaluated the theory of habitat sampling during the reproductive period using female wild turkeys as a model. We used high‐frequency location data gathered throughout the breeding and nesting period to evaluate the relationship between prenesting ranges, movements, and nest site selection of Rio Grande and Eastern wild turkeys in north‐central Texas and central Louisiana, respectively.

## Materials and Methods

### Study area

We collected data on Eastern wild turkeys (*Meleagris gallopavo silvestris*) on the Kisatchie National Forest (hereafter KNF) on the Kisatchie and Winn Ranger Districts in west‐central Louisiana, USA, from January to July 2014. The Kisatchie District comprised of 41,278 ha in Natchitoches Parish, whereas the Winn District comprises of 66,368 ha in Winn, Natchitoches, and Grant Parishes. Topography was rolling upland hills, high ridges, and sandy creek bottoms. The KNF was primarily composed of pine‐dominant forests, mixed pine‐hardwood forests, and hardwood‐dominant streams and drains; forest openings, utility right‐of‐ways, and forest roads were found throughout both ranger districts. Overstory trees included loblolly pine (*Pinus taeda*), shortleaf pine (*P. echinata*), longleaf pine (*P. palustris*), slash pine (*P. elliottii*), sweetgum (*Liqidambar styracaflua*), southern red oak (*Quercus falcata*), blackjack oak (*Q. marilandica*), post oak (*Q. stellata*), hickories (*Carya* spp.) and sweetbay (*Magnolia virginiana*). Understory plants included yaupon (*Ilex vomitoria*), American beautyberry (*Callicarpa americana*), broomsedge (*Andropogon virginicus*), panic grasses (*Panicum* spp. and *Dichanthelium* spp.), woodoats (*Chasmanthium laxum*), and brackenfern (*Pteridium aquilinum*). The US Forest Service typically conducted prescribed burns (both dormant and growing season) ranging in size from 7 to 1566 ha (18–22% of the available landscape) from December into May.

We collected data on Rio Grande wild turkeys (*Meleagris gallopavo intermedia*) from January 2012 to August 2014 in the Cross Timbers ecoregion in north‐central Texas, USA. Within the Cross Timbers, we conducted our work on private properties in Stephens and Palo Pinto counties and the Lyndon B. Johnson National Grasslands in Wise County. These sites consisted of rolling hills and steep canyons, with elevation from 122 to 518 m above sea level (Gould 1962). The region was predominately rangeland with various species of bluestem (*Andropogon* spp.), grama (*Bouteloua* spp.), and panicum (*Panicum* spp.), with common overstory species including live oak (*Quercus virginiana*), ashe juniper (*Juniperus ashei*), post oak (*Quercus stellata*), black jack oak (*Quercus marilandica*), and mesquite (*Prosopis glandulosa*). Cedar elm (*Ulmus crassifolia*), pecan (*Carya illinoinensis*), and cottonwood (*Populus deltoids*) were found along riparian areas. Study sites were managed for white‐tailed deer (*Odocoileus virginianus*) with other management strategies focused on Rio Grande wild turkey and northern bobwhite (*Colinus virginianus*).

### Capture and monitoring

We captured turkeys during January and February of 2012–2014 at baited sites using rocket nets (Dill 1969), drop nets (Glazener et al. 1964) and/or walk‐in traps (Davis 1994). All captured individuals were aged, sexed, and marked with an aluminum rivet leg band and fitted with a GPS‐VHF backpack‐style radio transmitter (Biotrack Limited, Wareham, UK) with the GPS programmed to record at 2‐ h increments from 0600 to 2000 daily with one location at midnight to identify roost site location. We used 1‐h sampling increments based on results from several studies (Byrne et al. 2015; Conley et al. 2015) and ongoing work evaluating the most appropriate sampling frequencies to describe space use by wild turkeys. Capture and handling protocols were approved by the Texas A&M University Institutional Animal Care and Use Committee (Permit 2010‐287) and the University of Georgia Institutional Animal Care and Use Committee (Permit A2013 12‐002‐Y1‐A0). We monitored all radio‐tagged individuals via radio‐telemetry weekly before the onset of nesting (approximately 1 April). We located radio‐tagged females >four times weekly during the nesting season (beginning approximately 15 March) so that nest location and initiation of incubation could be approximated by female movement patterns (Chamberlain and Leopold 2000; Collier et al. 2009). Upon suspected incubation, we located the general area of each nest site (visually locating without flushing) and continued to monitor the nesting female daily without disturbing the nest site to reduce researcher‐induced abandonment (Melton et al. 2011) until nest success or failure. We classified nest fate for our analysis as apparent success (i.e., hatching of ≥1 egg and locating a female with a brood) or failure via female absence at the nest ≥2 days, female mortality, or disposition of egg remains or lack or poults with the female immediately posthatch based on methods used wild turkey nest success studies (Dreibelbis et al. 2008, Collier et al. 2009; Melton et al. 2011; Conley et al. 2015).

### Statistical analysis

We incorporated each nest location and resultant movement data for each female during the prenesting, laying, and incubation period into ArcGIS 10.2 (Environment Systems Research Institute, Redlands, CA) database. We defined the prenesting period as the 45 days before the first record of an individual initiating a nest (Chamberlain and Leopold 2000). We defined the laying period based on hourly GPS movement data as the period during which the female made daily trips to the nest site for the purpose of laying, but typically roosted elsewhere in the immediate area (Collier and Chamberlain 2011). We defined the incubation period as beginning on the day the female approached the nest and all subsequent GPS locations were within a 50‐m buffer around the nest site for that day as found by Conley et al. (2015). Note we also cross‐validated the above delineations using our regular radio‐tracking data.

**Figure 1:**
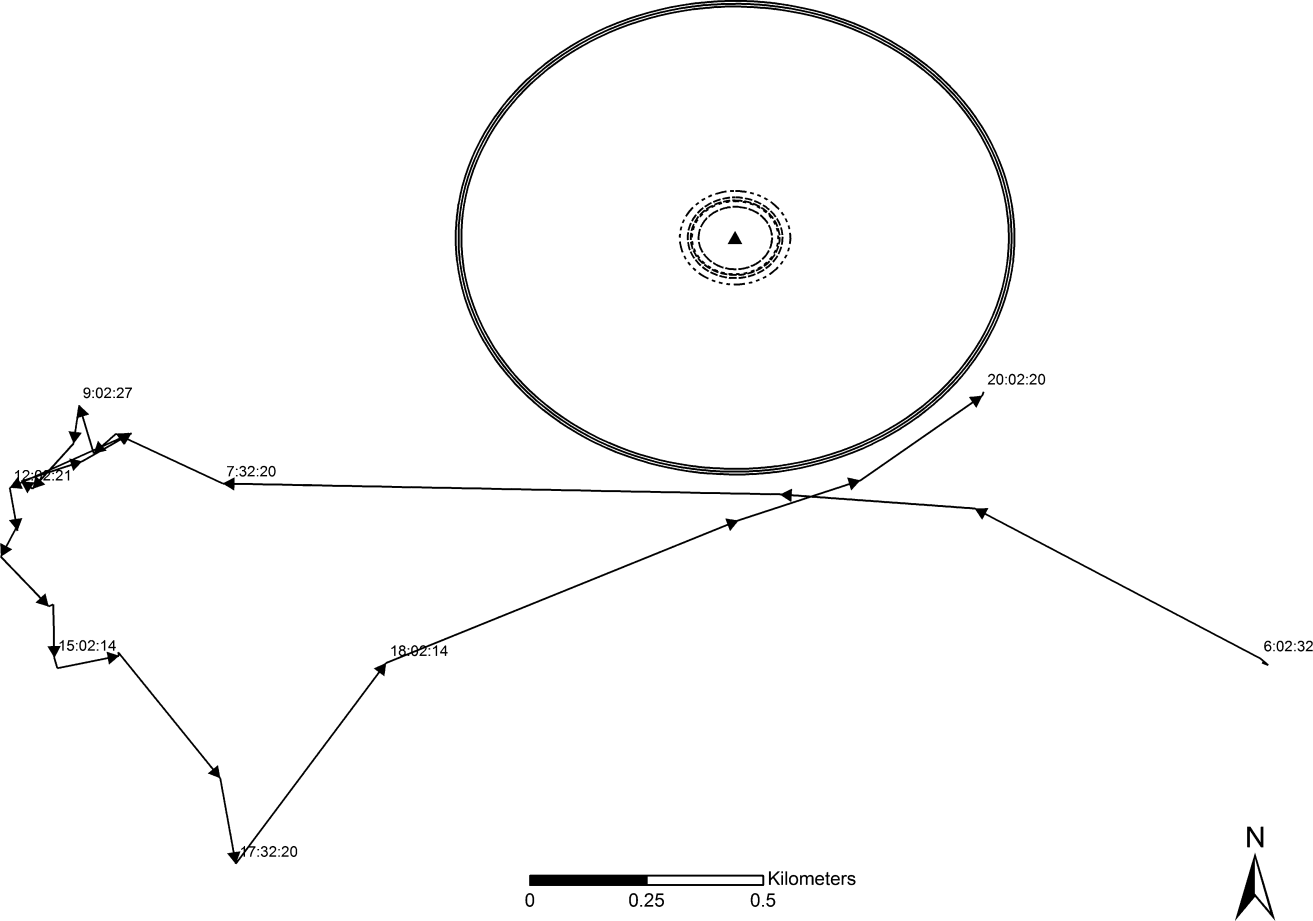
Example of individual‐specific, nest‐associated buffers showing no interaction during 1 day of movement for a Rio Grande wild turkey female relative to estimates of (1) the female's incubation range, (2) a 75% fixed kernel centered on the nest site, (3) the mean distance from the nest site, respectively, during incubation based on all females, and (4) 100 and 500 m buffers around each nest site. Using these buffers, we classified each hourly path segment as either intersected (1) or nonintersected (0) for regression analysis.

**Figure 2:**
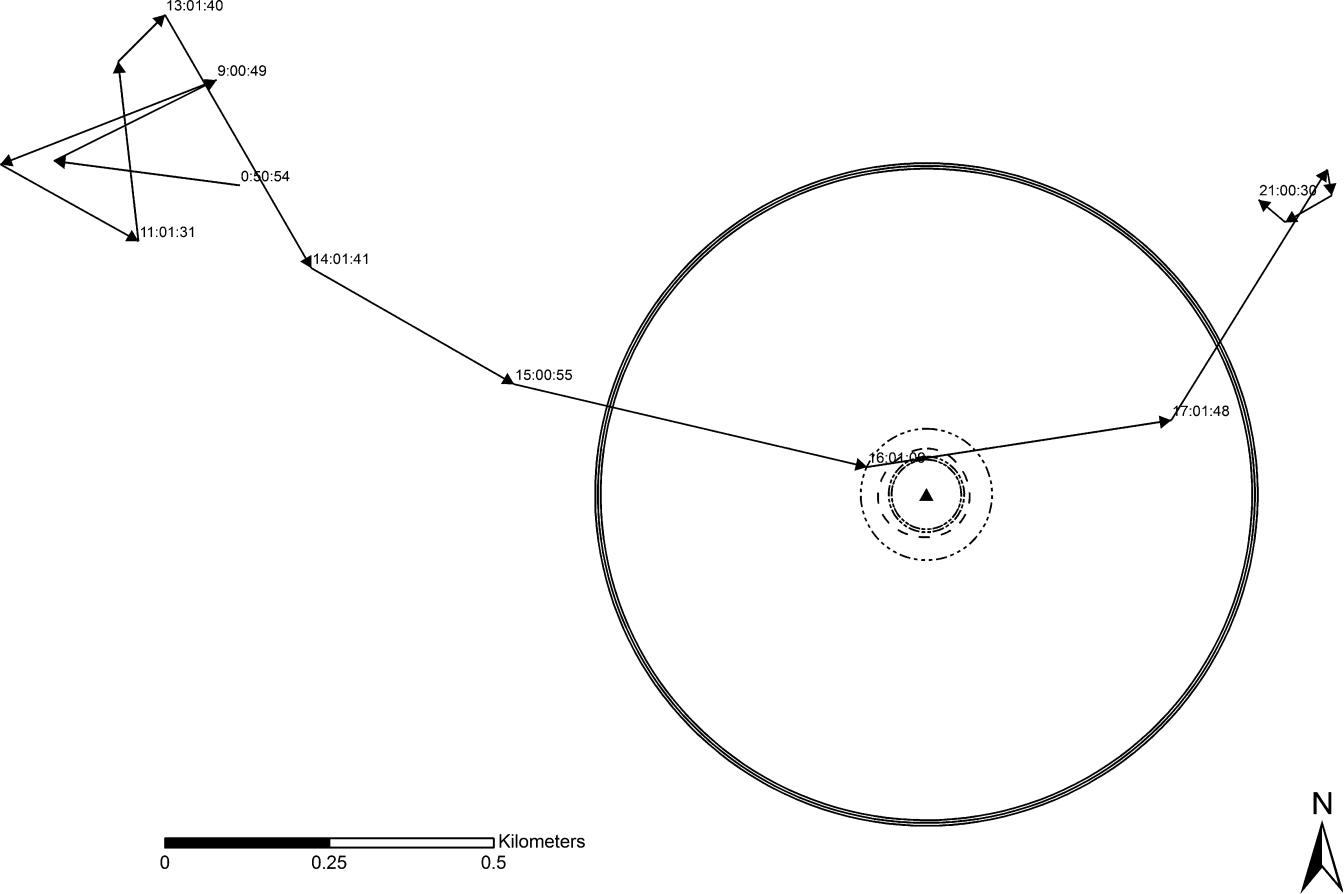
Example of individual‐specific, nest‐associated buffers showing multiple interactions during 1 day of movement for a Rio Grande wild turkey female relative to estimates of (1) the female's incubation range, (2) a 75% fixed kernel centered on the nest site, (3) the mean distance from the nest site, respectively, during incubation based on all females, and (4) 100 and 500 m buffers around each nest site. Using these buffers, we classified each hourly path segment as either intersected (1) or nonintersected (0) for regression analysis.

The theory of habitat sampling, and hence nest site selection, is founded on the expectation that the outcome of sampling and the selection process has demographic consequences (Jones 2001). Our work was not focused on demographic consequences of selection, but rather on whether the behavioral process of sampling was occurring, and whether that process could be identified based on movement information. Thus, first for each female, we calculated a prenesting range (50%, 95%, 99% kernels) based on GPS locations collected during the 45 days prior to incubation initiation using a dynamic Brownian bridge utilization distribution estimator (Kranstauber et al. 2012). Using the prenesting ranges, we evaluated whether or not a nest site location was within the associated core area (50% kernel), the typical estimate of a species range (95% kernel) and what we considered a maximum area of use by each individual (99% kernel). Next, for each female, we calculated the distance to each individual GPS location from the nest site each day and estimated the mean and minimum distance from the nest for all locations, by day, and for the entirety of the incubation period. Based on Guthrie et al. (2011), we excluded any GPS location that was estimated within a minimum distance of 10 m from the nest site to account for errors in GPS accuracy. This exclusion ensured we were not artificially biasing estimated movements low by including variation attributable to GPS location error when females were likely stationary while incubating their nests. We then used Hawth's Tools in ArcMap 9.3 (Beyer 2004) to construct 50%, 75%, and 99% fixed kernels for GPS locations collected from each female during the incubation period (Conley et al. 2015) and created individual‐specific, nest‐associated buffers of (1) 95% fixed kernel for the female's incubation range with a radius equating to the area of the kernel, (2) a 75% fixed kernel, (3) the mean distance from the nest site for all GPS location collected during incubation, respectively, during incubation based on all females (RGWT: 70.82 m, EWT: 55.24 m), and (4) 100 and 500 m buffers around each nest site (Conley et al. 2015; Figs 1 and 2). Using these buffers, we documented the frequency and distance for each hourly movement segment (connected linear path between two consecutive GPS locations) of the female's daily movement paths that intersected the boundary of each buffer described above for the 45 days before the female began laying (Tables 1 and 2). We classified each hourly path segment as either intersected (1) or nonintersected (0) and used logistic regression to model the probability that a female's movement path intersected the area (defined above) around the nest site location as a function of time (day) for each of the 45 days preceding the female laying her first egg and initiating a nest (Figs 1 and 2). We then used contingency table analysis to evaluate the odds of a female turkey having locations within the nest site buffer (smallest possible area detailed above) by comparing the frequency of locations 0–5 days before the female began laying to frequency of locations 6–15 days before laying, and then 6–15 to 15–45 days before laying began. We conducted all statistical analysis in R v3.1.3 (R Development Core Team 2015).

**Table 1:** Average and minimum distance moved from nest during the prenesting period for nesting Rio Grande wild turkeys in north‐central Texas, USA, during 2012–2013

Band ID	Days of prenest	Nest success/Fail	50%	Nest within prenest UD (range size in ha) 95%	99%	Mean distance (meters from nest) (SD)	Minimum distance to nest during prenesting (meters from nest)	Day closest to nest (days before laying)
8058	45	F	In (99.44)	In (599.71)	In (859.53)	385.82 (292.10)	6.47	3
A587(‘12)	33	F	Out (164.28)	Out (592.93)	Out (2760.9)	1149.99 (496.98)	355.37	18
A587(‘13)	36	F	Out (55.28)	In (850.06)	In (1347.6)	672.81 (419.41)	32.06	5
A590	45	F	Out (62.74)	In (669.23)	In (1121.2)	586.05 (430.03)	52.30	30
8009	44	F	Out (1170.7)	In (5437.83)	In (7461.24)	3267.51 (1459.30)	368.22	2
8013	45	F	Out (198.69)	In (920.86)	In (1338.1)	1931.40 (565.44)	24.07	2
8016	45	F	Out (102.96)	In (673.65)	In (1061.6)	829.89 (382.81)	30.22	7
8021	46	F	In (165.44)	In (597.1)	In (788.68)	755.88 (398.89)	3.51	2
8024	44	F	Out (169.19)	In (649.88)	In (852.21)	719.25 (501.32)	64.88	13
8026	45	F	Out (143.73)	In (595.19)	In (812.9)	689.30 (320.36)	109.37	12
8028	46	F	Out (178.09)	In (983.36)	In (1390)	1238.30 (598.61)	185.07	3
8029	43	F	In (1437.05)	In (3237.7)	In (20417.14)	6859.76 (4249.15)	36.04	12
21270	39	F	Out (299.19)	In (1772)	In (2780.9)	1149.74 (906.23)	24.03	2
8061	45	F	In (1844.2)	In (16251.03)	In (24134.74)	3452.15 (5812.75)	88.56	33
8063	45	F	Out (125.97)	In (1177.8)	In (1630.4)	3398.78 (1489.65)	2.77	2
8503	44	F	Out (72.38)	In (414.46)	In (619.38)	893.82 (470.09)	2.51	2
A588	45	S	Out (105.93)	In (520.24)	In (783.02)	702.86 (488.21)	29.73	4
A589	25	F	Out (60.97)	Out (291.09)	Out (447.92)	1301.07 (428.38)	662.18	3
A591	45	S	Out (116.69)	In (590.77)	In (924.31)	706.44 (456.13)	18.93	1
8134	41	F	Out (85.6)	In (625.05)	In (1036.4)	1563.82 (642.83)	139.50	3
8094	44	F	Out (104.99)	Out (658.12)	Out (971.9)	2102.48 (395.29)	1259.96	1
Mean	42.38		81% (Out)	14% (Out)	14% (Out)	1636.04	166.46	7.62
SD	5.21					1523.96	299.34	9.23

**Table 2:** Average and minimum distance moved from nest during the prenesting period for nesting Eastern wild turkeys in west‐central Louisiana, USA, during 2014

Band ID	Days of prenest	Nest success/Fail	50%	Nest within prenest UD (range size in ha) 95%	99%	Mean distance (meters from nest) (SD)	Minimum distance to nest during prenesting (meters from nest)	Day closest to nest (days before laying)
48	45	F	Out (157.44)	Out (1251.9)	Out (1506.5)	4231.81 (1283.99)	1359.08	3
50	45	F	Out (49.17)	In (427)	In (846.64)	384.35 (495.99)	31.04	6
51	46	F	Out (510.94)	In (3217.7)	In (4681)	4315.20 (2742.93)	156.31	4
132	45	F	In (429.72)	In (2050.2)	In (2846.2)	1890.91 (1986.24)	34.48	5
133	45	F	Out (115.05)	Out (433.37)	Out (603.28)	1210.52 (552.76)	367.19	5
134	45	F	Out (162.96)	Out (1109.5)	In (1610.6)	4502.91 (1550.50)	83.75	1
135	45	F	Out (489.29)	In (2347.9)	In (3573)	3424.63 (2359.39)	99.34	8
136	45	F	Out (230.73)	In (1079.8)	In (1556.1)	1349.59 (751.92)	192.39	7
369	45	F	In (341.75)	In (1625.8)	In (2235.4)	1101.39 (1132.95)	51.12	2
371	45	F	Out (274.24)	Out (1213.1)	In (1850.5)	1535.42 (734.1937)	503.43	9
372	45	F	Out (275.82)	In (1078.8)	In (1460.8)	1157.97 (701.54)	40.03	29
656	45	F	Out (532.92)	In (2090.1)	In (2790.9)	1678.61 (832.08)	4.95	2
657	45	F	Out (69.67)	In (610.64)	In (939.36)	778.53 (593.07)	112.53	1
658	45	F	Out (124.62)	In (757.84)	In (1084.3)	1259.55 (419.53)	205.37	7
660	45	F	In (269.76)	In (1010.6)	In (1409.7)	668.63 (378.85)	53.29	32
661	45	F	Out (85.32)	In (673.81)	In (1135.1)	1464.33 (513.21)	51.07	1
662	45	S	Out (36.99)	Out (284.86)	Out (423.65)	1511.46 (315.58)	824.96	44
663	45	F	Out (114.97)	In (849.53)	In (1305.7)	4208.09 (1408.03)	175.35	4
667	45	F	Out (311.37)	In (2012.5)	In (3008.9)	1626.79 (676.84)	112.42	44
710	45	F	Out (43.78)	Out (363.07)	In (620.35)	1904.47 (544.77)	86.46	2
777	45	F	Out (73.36)	In (380.65)	In (522.73)	621.78 (386.48)	<1	5
13210	45	F	Out (155.37)	Out (650.39)	Out (899.11)	1957.31 (645.75)	816.38	41
13181	45	S	Out (115.95)	In (694.57)	In (1045)	1776.36 (713.76)	73.90	4
Mean	45.04		87% (Out)	30% (Out)	13% (Out)	1937.42	235.01	11.57
SD	0.21					1267.84	337.90	14.72

## Results

We captured and GPS radio‐marked 21 Rio Grande and 23 Eastern wild turkeys during 2012–2014. The average number of days with available movement data during prenesting for Rio Grande and Eastern females was 42.38 (SE* *=* *5.21; range* *=* *25–46) and 45.04 (SE* *=* *0.21), respectively, and was dependent on the date captured relative to when laying began. For example, for a transmitter programmed to begin March 15, if a female began laying on April 15, then 30 days of prenesting movements would be available (Tables 1 and 2). Average daily distance of all locations from a nest site for Rio Grande and Eastern wild turkey females was 1636.04 m (SE* *=* *1523.96) and 1937.42 m (SE* *=* *1267.84), respectively, whereas average minimum distance of all locations from the nest site was 166.46 m (SE* *=* *299.34) and 235.01 m (SE* *=* *337.90), respectively (Tables 1 and 2). Most female Rio Grande (81%) and Eastern (87%) wild turkeys did not have nest sites within their core (50%) prenesting ranges, but most (85% RGWT and 82% EWT) fell within their 99% ranges (Tables 1 and 2).

During the 45‐day prenesting period, three Rio Grande females (14%) were closest to their nest between days 16 and 45, four (19%) between days 6 and 15, and 14 (67%) between days 1 and 5 (mean* *=* *day 8; range* *=* *33–1). For the 67% of Rio Grande females that had their minimum distance to the nest within ≤5 days before laying initiated, the average distance from the nest site was 210 m which increased during the ≤2‐day period slightly to 213 m (*t* = −0.012, df* *=* *12.2, *P* = 0.9899). The odds that a Rio Grande wild turkey female was located within the nest buffer during 1–5 days before laying was 2.9 (95% CI: 1.27–6.90) times the estimated odds of being located within their individual nest buffer during the 6‐ to 15‐day period. Odds of being located within the individual nest buffer 6–15 days before laying were 4.9 (95% CI: 1.85–13.88) times the estimated odds of being located within the nest buffer during the 16‐ to 45‐day period. For the 45‐day prenesting period, five Eastern females (22%) were closest to their nest between days 16 and 45, five (22%) between days 6 and 15, and 13 (56%) between days 1 and 5 (mean* *=* *day 12; range* *=* *44–1). For the 56% of Eastern wild turkey females that had their minimum distance to the nest within ≤5 days before laying initiated, the average distance from the nest site was 197 m which increased during the ≤2‐day period to 402 m (*t* = −1.06, df* *=* *12.1, *P* = 0.3089). The odds that an Eastern wild turkey female was located within the individual nest buffer during 1–5 days before laying were 5.2 (95% CI: 1.65–19.40) times the estimated odds of being located within the individual nest buffer during the 6‐ to 15‐day period. Odds of being located within the nest buffer 6–15 days before laying were 1.13 (95% CI: 0.31–3.43) times the estimated odds of being located within the nest buffer during the 16‐ to 45‐day period.

**Figure 3:**
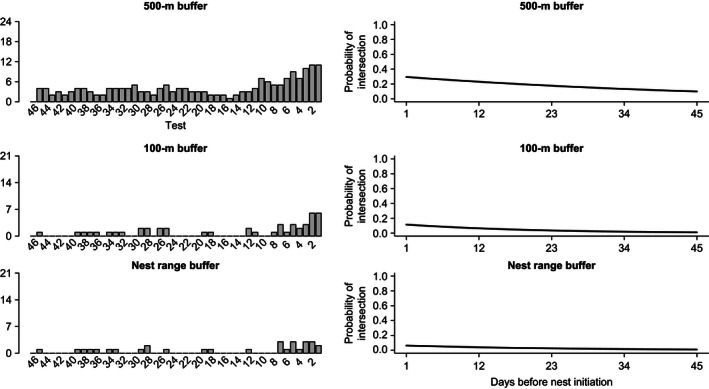
Left panel shows the relative frequency for Eastern wild turkey females (*n* = 23) interacting within a 500, 100 m, or individual‐specific nest range buffer relative to the number of days before laying was initiated. Right panel shows the logistic regression relationship estimating the probability that a female would be within the associated buffer size relative to days before laying began.

**Figure 4:**
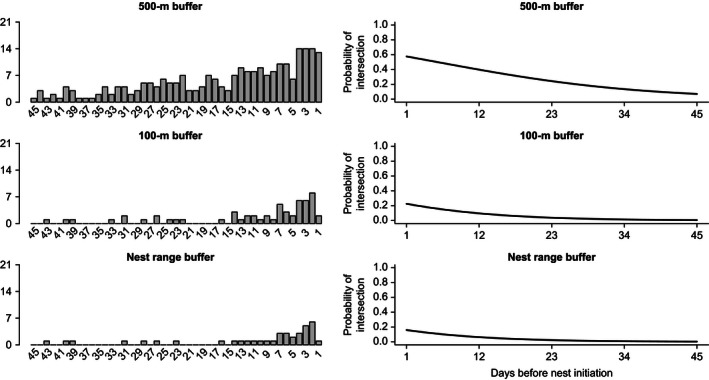
Left panel shows the relative frequency for Rio Grande wild turkey females (*n* = 21) interacting within a 500, 100 m, or individual‐specific nest range buffer relative to the number of days before laying was initiated. Right panel shows the logistic regression relationship estimating the probability that a female would be within the associated buffer size relative to days before laying began.

Both Rio Grande and Eastern wild turkey females had a greater probability (β_daysprenesting_ **=* *−0.06 and −0.03; *P* < 0.001) of their daily movements during prenesting intersecting with the largest of the nest area buffers, which increased as the first day of laying approached (500 m; Figs 3 and 4). The probability that a female was within 100 m of the nest site was predicted to be highest on the day before laying began (β_daysprenesting_ **=* *−0.09 and −0.05; *P* < 0.001), yet was < 0.25 for both subspecies. The estimated probability of intersection for all buffers approached zero as number of days before nest initiation increased. Likewise, the probability (β_daysprenesting_ **=* *−0.09 and −0.04; *P* < 0.001) that a female moved within the nest incubation range (75% kernel for incubation period) was < 0.20 for both subspecies (Figs 3 and 4).

## Discussion

Habitat selection is the process of behavioral responses that may result in the disproportionate use of areas that influence survival and fitness of individuals (Block and Brennan 1993; Jones 2001). Badyaev et al. (1996) suggested that selection would be manifested via (1) habitat sampling during the prenesting period allowing for selection of a better nest site, and (2) that selection should favor extended sampling by individuals, with (3) greater dispersal by higher‐fitness individuals being correlated with finding better quality sites early in the season, and (4) that the extent of habitat sampling early in the season also influenced reproductive performance by impacting renesting (see Jones 2001). Our findings suggest that the above assumptions and inferences that hinge on the basis that wild turkeys sample habitat before nesting as detailed by Badyaev et al. (1996) above may be erroneous. Hence, although the theoretical foundation has been that individuals should select habitat, and hence nest sites, that maximize reproductive success (Wiens 1989; Martin 1993; Jones 2001), we suggest that any demographic benefits may in fact not be driven by a habitat selection process, at least for wild turkeys. Based on our results, we suggest that the assumption of habitat sampling for nest site selection as purported by Badyaev et al. (1996) is incorrect.

Our focus was to determine whether habitat sampling was occurring during the period prior to nest initiation when turkeys have been assumed to be sampling (Badyaev et al. 1996; Chamberlain and Leopold 2000; Jones 2001; Collier and Chamberlain 2011). More than half (67% and 56%) of Rio Grande and Eastern wild turkey females, respectively, were closest to their future nest site ≤5 days before laying, and odds of being within the individual nest site buffer increased as nest initiation approached. Hence, if there is an appropriate temporal scale associated with the prenesting period, it should be tied to movements during the period immediately before laying, ≤5 or more likely ≤2 days. However, average minimum distances from each location to the nest site during prenesting exceeded 150 m (166 and 235 m for RGWT and EWT, respectively) and most females did not get within a minimum of 50 m from their nest site before the first egg was laid. Hence, it appears that most females do not sample areas within their ranges searching for specific nest sites before laying (Badyaev et al. 1996). We offer that a distance of 166 or 235 m away from eventual nest sites would encompass a wide variety of habitats and conditions in most systems, and is well outside the typical extent of area estimated as used (1.46 ha) by incubating females (Conley et al. 2015).

We recognize that wild turkeys could have sampled habitats outside of our monitoring period, perhaps during the wintering period preceding the nesting season. For example, the habitat sampling paradigm outlined by Badyaev et al. (1996) suggested that females choose a nest site before they choose a mate; hence, the amount of nesting habitat sampled is reflected by the extent of each female's movements and amount of area covered before initiating a nest. Our findings suggest this paradigm may be biologically implausible. Given the stochastic environment in which turkeys exist, the expectation that habitat or other environmentally driven conditions would be static over a time period of up to or exceeding 2 months is highly uncertain. For instance, our sample of Eastern wild turkeys existed on a landscape managed with extensive use (32,602 ha) of prescribed fires during the reproductive season (18–22% of the available landscape). Likewise, our sample of Rio Grande wild turkeys existed on a landscape driven by a drought–precipitation cycle occurring typically in March and April (Collier et al. 2009) with the bulk of nesting occurring in April through June (Melton et al. 2011). More importantly, our results provide no evidence that females search for and locate sites where their future nests occur during the prenesting period, as suggested by Badyaev et al. (1996) and Chamberlain and Leopold (2000). Within this vein, we acknowledge that there could be an experience aspect not evaluated here to nest site selection (Hoi et al. 2012), wherein individuals that nested in a particular location return to that location if successful in the preceding year. Potentially, our results could be impacted by memory, wherein individuals who previously have nested would have a memory of successful/unsuccessful locations. However, Locke et al. (2013) found for 194 Rio Grande wild turkey nesting attempts that distance between nest locations both within and between years were typically separated by over 1000 m and was unrelated to nest success or failure, we suggest that wild turkeys have low fidelity to nest locations, independent of previous demographic result, and thus memory is likely inconsequential.

Predation is the leading cause of mortality for most ground‐nesting birds (Chalfoun et al. 2002; Stephens et al. 2005), including wild turkeys (Miller and Leopold 1992). Predation risk can influence various aspects of bird behavior (Lima 2009), including fine‐scale foraging behavior (Suhonen 1993) and broader patterns of habitat use (Rodriguez et al. 2001). We recognize that predation risk could influence the behavior of female wild turkeys during prenesting periods, as females may select habitats within their ranges to minimize the risk of predation. If this occurred, then predation risk could have influenced our observations of female behavior prior to nest initiation. However, adequately assessing this potential was outside the scope of our work, as no accurate estimates of predator abundance or distribution existed on our study sites, and we lacked information detailing predator behavior as well. Regardless, female wild turkeys monitored in our study existed on two landscapes with relevant differences in vegetation, productivity, and community structure (Ames et al. 2016); hence, one would expect predation pressure to be variable across sites (Chalfoun et al. 2002). Because we observed similar behaviors across females in our study, we offer that predation risk is not likely the most important driver of female behavior during the period prior to nesting.

One area of continued interest in wildlife resource selection revolves around defining the appropriate scale of habitat selection (Orians and Wittenberger 1991). For wild turkeys, we suggest that evaluations of habitat selection should be redefined to the period immediately before nesting, as opposed to the ≥45‐day period previously defined by Chamberlain and Leopold (2000). Unfortunately, this time frame somewhat negates the basis of habitat sampling theory and use–availability models to identify or distinguish selection based on deviations in habitat use between nesting and nonnesting locations over time. Those methods, in theory, require some sort of sampling to occur. According to the definition provided by Jones (2001), the lack of hierarchical habitat selection, and the low probability that a wild turkey female uses areas within her eventual incubation range (Conley et al. 2015), suggest that perhaps nest site selection is a random event based on the physiological status of a reproductively capable individual rather than a process being driven by some underlying sampling regime.

## Conflict of Interest

None declared.
